# Tobacco and alcohol content in soap operas broadcast on UK television: a content analysis and population exposure

**DOI:** 10.1093/pubmed/fdaa091

**Published:** 2020-07-01

**Authors:** Alexander B Barker, John Britton, Emily Thomson, Rachael L Murray

**Affiliations:** Division of Epidemiology and Public Health, UK Centre for Tobacco and Alcohol Studies, City Hospital, University of Nottingham, , Nottingham NG5 1PB, UK

**Keywords:** alcohol consumption, public health, smoking

## Abstract

**Background:**

Exposure to tobacco and alcohol content in media is a risk factor for smoking and alcohol use in young people. Our previous research suggested that tobacco and alcohol imagery is common in soap operas. We now report an analysis of tobacco and alcohol content in a sample of soap operas broadcast in the UK.

**Methods:**

We used 1-minute interval coding to quantify tobacco and alcohol content in all episodes (including advertisement breaks) of six soap operas broadcast on UK television during three separate weeks in November and December 2018 and January 2019.

**Results:**

We coded 2222 intervals from 87 episodes and 360 intervals from 77 advertisement breaks. Tobacco content was rare, occurring in 4% of all intervals across 30% of episodes, the only tobacco appearances in adverts appeared in anti-smoking advertising. Alcohol occurred in 24% of intervals across 95% of episodes and in 13% of advertisement intervals. The programmes delivered ~381.28 million tobacco and 2.1 billion alcohol gross impressions to the UK population, including 18.91 million tobacco and 113 million alcohol gross impressions to children.

**Conclusion:**

Whilst tobacco was rare, alcohol content was common, resulting in billions of viewer impressions. Soap operas represent a potential driver of alcohol consumption in young people.

## Introduction

Smoking and alcohol consumption increases the risk of developing serious health conditions, including cancer, stroke and heart disease.^1^^,^^2^ In 2017–18, smoking and alcohol consumption, respectively, caused an estimated 78 thousand^3^ and 7.5 thousand deaths^4^ in the UK. In the UK alone, there were 485 and 338 thousand hospital admissions due to tobacco and alcohol, respectively,^5^^,^^6^ imposing a financial burden of £6 billion on the National Health Service and a substantially greater financial and amenity loss on wider society.^7–9^ Since almost all adults who smoke begin smoking during teenage years,^10^ and alcohol consumption in adolescence is associated with a higher risk of consumption in adulthood,^11^ it is important to prevent children and adolescents from experimenting with these behaviours.

There is now strong evidence that exposure to tobacco and alcohol imagery in the media, whether as programme content or commercial advertising, increases tobacco and alcohol use by adolescents.^12–20^ Tobacco and alcohol content in the media normalizes these behaviours for young people, and young people may imitate behaviours of influential others, such as celebrities.^21–23^ An estimated 28.5 million UK homes have a television,^24^ and UK citizens typically consume more than 3 hours of television and 25 minutes of subscription video-on-demand each day.^25^ Television programme content that is potentially harmful to children, including tobacco and alcohol content, is regulated by the Office of Communications (Ofcom) Broadcasting Code^26^ (Section 1.10), which prohibits depictions of alcohol and tobacco use in programmes made for children or, in the absence of editorial justification, in programmes made for wider audiences, broadcast before the 9 p.m. watershed and likely to be widely seen, heard or accessed by those aged under 18.^27^ Television advertising content is regulated by the Advertising Standards Authority (ASA), and whilst paid-for tobacco advertising and product placement should no longer occur, having been prohibited in the UK since 1965,^28^ for alcohol the ASA broadcast advertising code states only that advertisements should not ‘be likely to appeal strongly to people under 18, especially by reflecting or being associated with youth culture or showing adolescent or juvenile behaviour’ and alcohol advertisements ‘may not be advertised in or adjacent to programmes commissioned for, principally directed at or likely to appeal particularly to audiences below the age of 18’.^29^ However, this does not prevent young viewers being exposed to this content whilst watching programmes, which are not commissioned for them.

Soap operas (soaps) have been a staple of UK prime-time television broadcasting for decades, with current programmes such as *EastEnders*, *Coronation Street* and *Emmerdale* averaging nearly 7 million viewers per episode.^30^ Soaps are often made to attract young viewers,^31^^,^^32^ are popular with young people^33^^,^^34^ and are regularly watched by children with their family.^35^^,^^36^ Since soaps are made to reflect daily life, they include tobacco and alcohol consumption, hence potentially promoting these behaviours among children.^35^ We have therefore quantified the extent of tobacco and alcohol content in soaps broadcast on UK television in 2018/2019 and estimated the child exposure generated.

## Methods

Six of the most popular UK soaps shown on the five national UK free-to-air channels were selected (*EastEnders* British Broadcasting Corporation (BBC1), *Coronation Street* Independent Television Channel (ITV), *Emmerdale* [ITV], *Hollyoaks* [Channel 4], *Neighbours* [Channel 5], *Home and Away* [Channel 5]). Two of these, *Neighbours* and *Home and Away*, are produced in Australia, the remainder in the UK. The programmes (including advertisement breaks in the middle of programmes, either advertisements for other programmes only, as seen in *EastEnders* or other programmes, or commercial adverts, as seen in *Coronation Street*, *Emmerdale*, *Hollyoaks, Neighbours* and *Home and Away*) were viewed and coded over a period of three separate weeks (Monday to Sunday) in 2018/2019, with a 4-week gap between each (5 November–11 November, 3 December–9 December, 7 January–13 January). Every soap opera featured in this study was broadcast before the Ofcom 9 p.m. watershed, the time at which TV programmes unsuitable for children can be broadcast.^27^ All footage, including advertisement breaks, was viewed and semi-quantitatively coded in 1-minute intervals as used extensively in previous studies.^37–43^ The method involves recording the presence or absence of audio-visual content in every 1-minute period using the following categories:

*Actual use*: Use of tobacco or alcohol onscreen by any character, such as actually smoking or consuming alcohol in a scene.

*Implied use*: Any inferred tobacco or alcohol use without any actual use on screen, such as a character holding a cigarette or drink but without actually seeing them consume or smoke it.

*Other tobacco/alcohol reference*: The presence onscreen of tobacco or alcohol or related materials, such as cigarette packets or bottles.

*Brand appearance*: The presence of clear and unambiguous tobacco or alcohol branding, such as branding seen on marketing materials or bottles.

We also recorded appearance of electronic cigarettes but, after seeing only one appearance, excluded electronic cigarettes from further analysis.

Tobacco and alcohol content were recorded as present in the 1-minute interval if there was one appearance of any category in that interval. More than one category could be coded in a single interval, for example, both alcohol and tobacco use, but multiple instances of the same category in the same interval were recorded as a single event. If the same event overlapped two intervals, this was coded as two separate events. One-third of the recorded footage was coded separately by two authors to ensure accuracy and reliability in the coding method. Coding was completed using Microsoft Excel and, on completion, data were entered into IBM SPSS Statistics 24 for analysis.

We estimated UK audience exposure using viewing data from *Digital-i*^44^ and used UK mid-year population estimates for 2017^45^ combined with numbers of tobacco and alcohol appearances to estimate gross and per capita impressions by age group, using previously reported methods.^40^^,^^46^^,^^47^ Viewership was combined with the number of tobacco and alcohol appearances per episode to provide gross impressions. Per capita impressions were calculated by dividing gross impressions by population estimates. Analyses were conducted in IBM SPSS Statistics (V.24) and Microsoft Excel (2013). The confidence level was set to 95%.

## Results

In total 87 soap episodes were included in the study, as well as 77 advertisement breaks. Further details on the sample of broadcasts can be found in Table 1.

**Table 1:** Information on the sample of programmes included in the study

	*Number of episodes/broadcasts*	*Number of soap intervals*	*Number of advertisement breaks*	*Number of advertisement break intervals*
Total	87	2222	77	360
*EastEnders*	12	345	0	0
*Coronation Street*	18	447	18	87
*Emmerdale*	18	464	20	99
*Hollyoaks*	14	358	14	63
*Home and Away*	15	368	15	68
*Neighbours*	10	240	10	43

### Tobacco

Tobacco content occurred in 85 intervals (4%) in 26 soap episodes (30%), with actual use occurring in two intervals. Other tobacco references were seen in 83 intervals across 26 episodes and mostly involved no smoking signs, which occurred in 70 intervals (84% of paraphernalia intervals). Cigarette packets, all of them plain, were seen in four intervals (5% of paraphernalia intervals), with cigarette lighters occurring three intervals and an ashtray and matches in one interval each. Implied tobacco use occurred in four intervals, two of which were verbal and two non-verbal. No tobacco branding was seen. Most^52^ tobacco intervals occurred in ITV broadcasts, with *Coronation Street* containing 46 of them. There was no tobacco content in *Hollyoaks*, *Home and Away* or *Neighbours*. The non-commercial channel (BBC1) contained significantly more episodes containing tobacco content (*P* < 0.001) and intervals containing tobacco content (*P* < 0.05) than non-commercial channels (ITV, Channel 4 and Channel 5).

We estimate that the 87 soap opera episodes delivered 381.28 million tobacco gross impressions (95% CI 359.33–403.24) to the UK population, including 18.91 million (95% CI 16.74–21.08) to children aged under 16. Tobacco impressions per capita were highest (average 11.65 (95% CI 11.20–12.10) in the 65+ age group. Children received on average 1.50 (95% CI 1.33–1.68) per capita impressions. Women received on average more per capita impressions (average 7.53 (95% CI 7.15–7.91) than men (average 3.89 (95% CI 3.6–4.15). For a breakdown of tobacco gross and per capita impressions per episode, see Supplementary File 1. Removing ‘no smoking signs’ from our analysis revealed that tobacco content occurred in 9 episodes (10.34%). This delivered ~70.3 million tobacco impressions to the UK population, including 4.2 million to children aged under 16.

Tobacco content occurred in only two 1-minute intervals during advertisements, both during anti-smoking advertising by Public Health England and an advert for nicotine replacement therapy.

### Alcohol

**Fig. 1:**
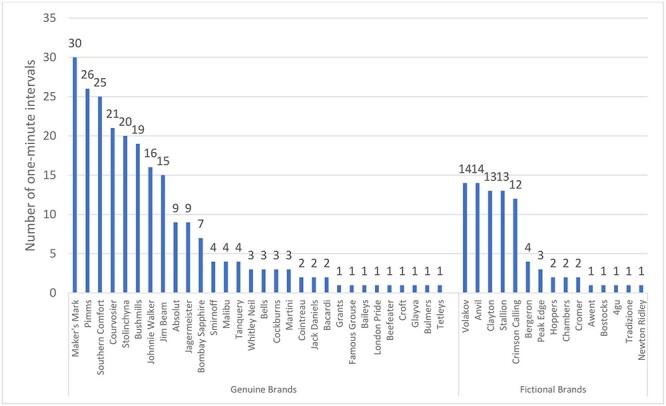
Occurrence of genuine and fictional alcohol brands in UK soap operas.

Alcohol content was seen in 526 (24%) intervals in 83 (95%) soap episodes. Actual alcohol use occurred in 109 intervals (21% of alcohol content intervals) across 48 episodes (57% of episodes containing alcohol content), most frequently comprising consumption of beer or cider (50 intervals, 46% of alcohol use intervals). Just over half of alcohol consumption was by women (58 intervals, 53% of alcohol use intervals). Three intervals contained alcohol use by under 18s (3% of alcohol use intervals). Alcohol paraphernalia was seen in 390 intervals (18% of all intervals) across 80 episodes (92% of all episodes) and mostly involved beer pumps or bottles (324 intervals, 83% of all paraphernalia intervals). Implied alcohol use was seen in 311 intervals (14% of all intervals) across 71 episodes (82% of episodes), with people holding a drink being the most common occurrence (275 intervals, 88% of implied use intervals). Alcohol branding was seen in 107 intervals (5% of all intervals) across 37 episodes (43% of episodes), occurring exclusively through beer pumps or labels on bottles in the background of scenes. Overall, 45 brands were seen, of which 30 were genuine brands and 15 fictional (Fig. 1). Genuine brands were often placed side by side with fictional brands.

The channel broadcasting the most alcohol content was ITV (277 intervals, 30% of intervals broadcast on ITV), with Channel 4 broadcasting the least (53 intervals, 15% of intervals broadcast on Channel 4). *Coronation Street*, broadcast on ITV, contained the most intervals (159 intervals, 36% of *Coronation Street* intervals) and *Hollyoaks*, broadcast on Channel 4, the least (53 intervals, 15% of *Hollyoaks* intervals). Commercial channels contained significantly more episodes containing alcohol content than on the non-commercial channel (*P* < 0.05); however no significant difference was found in the number of intervals containing alcohol content shown in soaps broadcast on commercial and non-commercial channels.

**Fig. 2:**
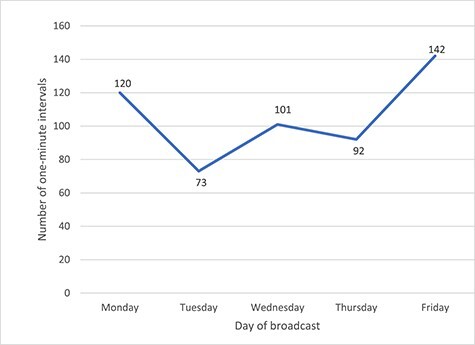
Occurrence of 1-minute intervals containing alcohol content by day of broadcast.

Alcohol content occurred more frequently in programmes broadcast in November than in December and January (201, 172 and 153 intervals, respectively) and was also more likely to appear on Fridays than other weekdays (Fig. 2), though these differences were not statistically significant.

During advertisement breaks, alcohol content was seen in 46 intervals (13% of advertisement break intervals), with branding in 13 (28% of advertisement intervals containing alcohol content). The most common brand seen in adverts were *Jack Daniels*, *The Famous Grouse*, *Disaronno* and *Chambord* (all two intervals each). Alcohol branded adverts were only seen in November and December.

### Differences in country of origin

**Fig. 3:**
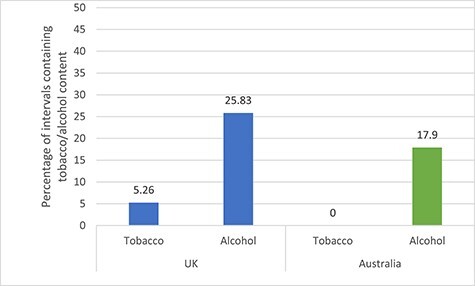
Percentage of 1-minute intervals containing tobacco and alcohol content by country of origin.

The proportion of intervals containing tobacco and alcohol content differed by the country in which the programme was made. *Home and Away* and *Neighbours*, which are produced in Australia, contained no tobacco imagery and a lower proportion of alcohol intervals than soaps made in the UK (see Fig. 3). Genuine branded products were only seen in UK soaps.

We estimate that the 87 soap episodes delivered 2.1 billion alcohol gross impressions (95% CI 1.9–2.2) to the UK population, including 113 million (95% CI 99–127) to children aged under 16. Alcohol impressions per capita were highest (average 64.30, 95% CI 61.51–67.07) in the 65+ age group. Children received an average of 9.13 (95% CI 7.99–10.28) impressions per capita, and women received more per capita impressions (average 42.11 (95% CI 39.75–44.47) than men (average 22.21 (95% CI 20.45–23.95). There were 568 million (95% CI 534–602) gross impressions of branded alcohol products, including 26.62 million to children (95% CI 23.31–29.92). For a breakdown of alcohol gross and per capita impressions per episode, see Supplementary File 2.

## Discussion

### Main findings of the study

This study demonstrates that in soaps broadcast on UK television in winter 2018–19, tobacco imagery was rare but alcohol imagery occurred in almost every episode examined, confirming an earlier observation^37^ that soap opera TV programmes in the UK are a significant source of exposure of children alcohol imagery. There were also numerous alcohol brand appearances, with genuine alcohol branding being common in soaps shown on UK television before the 9 p.m. watershed.^27^ The low frequency of tobacco imagery the tobacco content shown in these programmes generated substantial population exposure, including millions of tobacco impressions, but these arose primarily from no-smoking signs and are unlikely to lead to appreciable harm. However, the alcohol content generated billions of impressions, including branding imagery from real alcohol products, to children aged under 16.

The programmes selected for this study represent a range of long running soaps shown on UK television, all of which are shown before the 9 p.m. watershed,^27^ all likely to be viewed as part of family viewing,^35^^,^^36^ and all therefore subject to Ofcom regulations intended to protect children from alcohol imagery.^27^ Children watching these programmes also encountered substantial alcohol imagery in paid-for advertising breaks during the programmes. Our findings demonstrate therefore that current legislation prohibiting tobacco advertising and product placement in television appears to be effective, with no episodes of tobacco branding seen in these programmes but that, in contrast, alcohol regulations are failing to prevent a substantial degree of generic and branded exposure.

Soap operas have traditionally been made to reflect daily life,^48^ and British soaps have typically involved a local pub^49^ and therefore contain many scenes depicting alcohol. This is particularly so on Fridays, the end of the working week for many people, and hence a day on which alcohol is more likely to be consumed. It is presumably this that provides the editorial justification required by Ofcom^27^ to justify including alcohol imagery. However the concern remains that although grounded in reality with the potential to provide ‘educational entertainment’^50^ capable of changing social norms and attitudes,^50^^,^^51^ it is likely that the high occurrence of alcohol use will drive alcohol consumption among young people. In this respect therefore, British soap operas are likely to be contributing to the normalization of drinking behaviours in young and future generations. This is especially so in relation to branding, with these programmes delivering ~600 million branded alcohol impressions to the UK population, including 26 million to children under the age of 16. Whilst the Ofcom broadcasting code^52^ prohibits paid-for alcohol product placement, programme makers can use ‘props’, items which they do not receive payment for using,^53^ or fictional brands. We noticed that genuine brands were shown in programmes alongside fictional brands, calling into question why genuine alcohol brands are being used in scenes. Furthermore, according to YouGov ratings, a measure of popularity and fame based on millions of responses from the British public,^54^ many of the brands featured in these programmes are not popular in the UK, with the most prominent brand, Maker’s Mark, not appearing in the YouGov list.^55^ The inclusion of these brands in UK soap operas is thus unjustifiable on the grounds of reflecting everyday life.

Our results suggest that the commerciality of a channel may affect the content shown in that, more alcohol content was shown on commercial channels and more tobacco content was shown on the soap on the non-commercial channel. Branded alcohol advertisements were shown during soap opera advertisement breaks on commercial channels, exposing young viewers to branded alcohol content. However, these advertisements comply with the ASA broadcast advertising code as soap operas are not commissioned for or principally aimed at audiences below the age of 18.

The current study included two soaps, *Neighbours* and *Home and Away*, which are produced in Australia. Both contained significantly less alcohol and tobacco content than their UK alternatives. These differences could be due to differences in regulation. In Australia, all programmes broadcast on television are classified as suitable for certain ages, with the age rating affecting when a programme can be broadcast, with material that is suitable for those 15 and over only being broadcast after 7:30 p.m. All programmes broadcast before this must be classified as PG or below. The PG guidance states that ‘the use of legal drugs must be handled with care’.^56^ Australian soaps are broadcast in the early evening and therefore must abide by the PG guidance.

### What is already known on this topic

Experimentation and initiation of smoking and alcohol use during adolescence is a strong risk factor for dependence and continued use in later life. There is strong evidence that exposure to tobacco and alcohol imagery in the media increases experimentation and use in young people. Previous studies have shown that tobacco and alcohol content is regularly shown in soap operas broadcast on UK television.

### What this study adds

Tobacco and alcohol content shown in the media has an effect on the experimentation and uptake of smoking and alcohol use in young people. Our analysis shows that whilst tobacco content was rare, soap operas shown on UK television are a major source of exposure to alcohol content for young people and is likely to be a potential driver for alcohol use. The Ofcom broadcasting code protects under 18s from tobacco and alcohol content by restricting depictions of tobacco and alcohol use in programmes made for children and discouraging the use of tobacco or alcohol in programmes broadcast before the 9 p.m. watershed or otherwise likely to be widely seen, heard or accessed by children. The current study shows that soap operas, which were all broadcast before the 9 p.m. watershed, are widely seen and accessed by young people. Whilst current legislation prohibiting tobacco advertising and product placement in television appears to be effective, with no episodes of tobacco branding seen in these programmes and the majority of content consisting of ‘no smoking’ signs, the amount of alcohol content and genuine branding featured in soap operas is alarming and is widely seen by young viewers. The Ofcom 9 p.m. watershed, designed to protect children and young people from potentially harmful imagery, is not fulfilling its purpose in relation to soap operas. Tighter scheduling rules, such as showing these programmes after the 9 p.m. watershed,^27^ restricting alcohol advertisements being shown before the 9 p.m. watershed or else following the example of the Australian soap operas and reducing the reliance on alcohol imagery, could prevent children and adolescents being exposed to this content. Ofcom should investigate the use of genuine brands in soaps to ensure that the use of these products complies with the broadcasting code.^26^ Future studies should continue to monitor alcohol and tobacco content throughout the year to determine the true exposure to this content.

## Limitations of this study

Whilst we explored soaps broadcast on free-to-air UK television, British soaps are broadcast and popular around the world, and the true exposure from this content could be greater. We acknowledge that soap operas are broadcast throughout the year and that storylines may change seasonally. We explored content over a period of 3 months, which included the run up to Christmas and the New Year period. Alcohol content during advertisements and programmes was higher before than after the Christmas and New Year holidays. Whilst there is currently no evidence of seasonal variation in the amount of tobacco and alcohol content shown in soap operas, we acknowledge that the lack of alcohol content in January could be due to the cultural reflection of alcohol abstinence due to New Year’s Resolutions; however, this was not explored in any storylines. Similarly, the amount of content, and the impressions resulting from this content, could potentially be higher in the current study than at other times of the year due to the lead up to Christmas being reflected in the storylines. Further research should investigate whether there are any seasonal variations, for example, whether content differs between summer and winter.

We used interval coding methods to generate semi-quantitative measures of content over a standardized period of time to allow direct comparison between programmes, which are shown for different amounts of time, therefore allowing an exploration of the percentage proportion of a programme. This method can lead to both underestimation (if high-frequency appearances are concentrated in short periods of time) and overestimation (if short appearance transition into two intervals) and has been widely used in previous studies.^37–42^^,^^46^^,^^57–63^ Alternative approaches such as frequency analysis,^64–68^ whereby all visual appearances are counted as individual events irrespective of duration, are available but assume that a single long appearance carries the same impact as a short appearance.

## Supplementary Material

soaps_sup1_tob_fdaa091

soaps_sup2_alc_fdaa091

## Notes

**Alexander B. Barker,** Research Fellow

**John Britton,** Professor of Epidemiology; Director, UK Centre for Tobacco & Alcohol Studies

**Emily Thomson,** Medical Student

**Rachael L. Murray,** Associate Professor in Health Policy
