# Corrigendum to: Tobacco and alcohol content in soap operas broadcast on UK television: a content analysis and population exposure

**DOI:** 10.1093/pubmed/fdaa133

**Published:** 2020-07-30

**Authors:** Alexander B Barker, John Britton, Emily Thomson, Rachael L Murray

**Affiliations:** Division of Epidemiology and Public Health, UK Centre for Tobacco and Alcohol Studies, City Hospital, University of Nottingham, Nottingham NG5 1PB, UK

In the original article, the following was accidently omitted from the acknowledgements section: JB and RLM are members of SPECTRUM a UK Prevention Research Partnership Consortium. UKPRP is an initiative funded by the UK Research and Innovation Councils, the Department of Health and Social Care (England) and the UK devolved administrations, and leading health research charities. This has now been corrected. The title of Emily Thomson was also missing from the author designation box section. Her title of Medical Student has now been added.

