# Supplementary material for: Tobacco and alcohol content in soap operas broadcast on UK television: a content analysis and population exposure
Source: J Public Health (Oxf). 2020 Jul 1;43(3):595–603. doi: 10.1093/pubmed/fdaa091 (PMC8458014; doi:10.1093/pubmed/fdaa091)
Supplement: soaps_sup1_tob_fdaa091 [file soaps_sup1_tob_fdaa091.docx]

| Episode | | November | | | | | | | | | | December | | | | | | | | | | January | | | | | | | | | |
| --- | --- | --- | --- | --- | --- | --- | --- | --- | --- | --- | --- | --- | --- | --- | --- | --- | --- | --- | --- | --- | --- | --- | --- | --- | --- | --- | --- | --- | --- | --- | --- |
|  |  | 5^th^ Part 1 | 5^th^ Part 2 | 6^th^ Part 1 | 6^th^ Part 2 | 7^th^ Part 1 | 7^th^ Part 2 | 8^th^ Part 1 | 8^th^ Part 2 | 9^th^ Part 1 | 9^th^ Part 2 | 3^rd^ Part 1 | 3^rd^ Part 2 | 4^th^ Part 1 | 4^th^ Part 2 | 5^th^ Part 1 | 5^th^ Part 2 | 6^th^ Part 1 | 6^th^ Part 2 | 7^th^ Part 1 | 7^th^ Part 2 | 7^th^ Part 1 | 7^th^ Part 2 | 8^th^ Part 1 | 8^th^ Part 2 | 9^th^ Part 1 | 9^th^ Part 2 | 10^th^ Part 1 | 10^th^ Part 2 | 11^th^ Part 1 | 11^th^ Part 2 |
| Eastenders | Proportion viewership (000s) | 0.096 |  | 0.098 |  |  |  | 0.096 |  | 0.092 |  | 0.093 |  | 0.099 |  |  |  | 0.093 |  | 0.089 |  |  |  | 0.086 |  |  |  | 0.101 |  | 0.097 | 0.082 |
|  | Gross Impressions (Million) | 6.355 |  | 6.490 |  |  |  | 25.463 |  | 18.423 |  | 6.183 |  | 0 |  |  |  | 6.171 |  | 5.923 |  |  |  | 0 |  |  |  | 6.767 |  | 6.419 | 5.504 |
|  | Per Capita Impressions | 0.096 |  | 0.098 |  |  |  | 0.383 |  | 0.277 |  | 0.093 |  | 0 |  |  |  | 0.093 |  | 0.089 |  |  |  | 0 |  |  |  | 0.102 |  | 0.097 | 0.083 |
|  |  |  |  |  |  |  |  |  |  |  |  |  |  |  |  |  |  |  |  |  |  |  |  |  |  |  |  |  |  |  |  |
| Coronation Street | Proportion Viewership (000s) | 0.089 | 0.085 |  |  | 0.085 | 0.078 |  |  | 0.090 | 0.086 | 0.082 | 0.083 |  |  | 0.080 | 0.074 |  |  | 0.081 | 0.079 | 0.086 | 0.082 |  |  | 0.084 | 0.082 |  |  | 0.086 | 0.083 |
|  | Gross Impressions (Million) | 35.464 | 11.298 |  |  | 22.496 | 15.517 |  |  | 5.994 | 0 | 5.445 | 27.656 |  |  | 0 | 0 |  |  | 0 | 10.501 | 22.864 | 0 |  |  | 33.455 | 32.711 |  |  | 22.840 | 11.229 |
|  | Per Capita Impressions | 0.534 | 0.170 |  |  | 0.339 | 0.234 |  |  | 0.090 | 0 | 0.082 | 0.417 |  |  | 0 | 0 |  |  | 0 | 0.158 | 0.344 | 0 |  |  | 0.504 | 0.492 |  |  | 0.344 | 0.169 |
|  |  |  |  |  |  |  |  |  |  |  |  |  |  |  |  |  |  |  |  |  |  |  |  |  |  |  |  |  |  |  |  |
| Emmerdale | Proportion Viewership (000s) | 0.078 |  | 0.074 |  | 0.075 |  | 0.072 | 0.069 | 0.068 |  | 0.073 |  | 0.071 |  | 0.075 |  | 0.070 | 0.070 | 0.070 |  | 0.075 |  | 0.073 |  | 0.074 |  | 0.072 | 0.074 | 0/078 |  |
|  | Gross Impressions (Million) | 0 |  | 9.775 |  | 9.998 |  | 0 | 0 | 10.335 |  | 0 |  | 0 |  | 0 |  | 0 | 0 | .0 |  | 0 |  | 0 |  | 0 |  | 0 | 0 | 0 |  |
|  | Per Capita Impressions | 0 |  | 0.147 |  | 0.151 |  | 0 | 0 | 0.156 |  | 0 |  | 0 |  | 0 |  | 0 | 0 | 0 |  | 0 |  | 0 |  | 0 |  | 0 | 0 | 0 |  |
|  |  |  |  |  |  |  |  |  |  |  |  |  |  |  |  |  |  |  |  |  |  |  |  |  |  |  |  |  |  |  |  |
| Hollyoaks | Proportion Viewership (000s) | 0.012 |  | 0.011 |  | 0.013 |  | 0.014 |  | 0.013 |  |  |  | 0.012 |  | 0.010 |  | 0.012 |  | 0.010 |  | 0.013 |  | 0.014 |  | 0.013 |  | 0.015 |  | 0.012 |  |
|  | Gross Impressions (Million) | 0 |  | 0 |  | 0 |  | 0 |  | 0 |  |  |  | 0 |  | 0 |  | 0 |  | 0 |  | 0 |  | 0 |  | 0 |  | 0 |  | 0 |  |
|  | Per Capita Impressions | 0 |  | 0 |  | 0 |  | 0 |  | 0 |  |  |  | 0 |  | 0 |  | 0 |  | 0 |  | 0 |  | 0 |  | 0 |  | 0 |  | 0 |  |
|  |  |  |  |  |  |  |  |  |  |  |  |  |  |  |  |  |  |  |  |  |  |  |  |  |  |  |  |  |  |  |  |
| Neighbours | Proportion Viewership (000s) | 0.011 |  | 0.011 |  | 0.012 |  | 0.011 |  | 0.008 |  | 0.011 |  | 0.010 |  | 0.010 |  | 0.010 |  | 0.011 |  | 0.012 |  | 0.012 |  | 0.010 |  | 0.010 |  | 0.011 |  |
|  | Gross Impressions (Million) | 0 |  | 0 |  | 0 |  | 0 |  | 0 |  | 0 |  | 0 |  | 0 |  | 0 |  | 0 |  | 0 |  | 0 |  | 0 |  | 0 |  | 0 |  |
|  | Per Capita Impressions | 0 |  | 0 |  | 0 |  | 0 |  | 0 |  | 0 |  | 0 |  | 0 |  | 0 |  | 0 |  | 0 |  | 0 |  | 0 |  | 0 |  | 0 |  |
|  |  |  |  |  |  |  |  |  |  |  |  |  |  |  |  |  |  |  |  |  |  |  |  |  |  |  |  |  |  |  |  |
| Home and Away | Proportion Viewership (000s) | 0.009 |  | 0.008 |  | 0.008 |  | 0.008 |  | 0.008 |  |  |  |  |  |  |  |  |  |  |  | 0.009 |  | 0.009 |  | 0.008 |  | 0.008 |  | 0.008 |  |
|  | Gross Impressions (Million) | 0 |  | 0 |  | 0 |  | 0 |  | 0 |  |  |  |  |  |  |  |  |  |  |  | 0 |  | 0 |  | 0 |  | 0 |  | 0 |  |
|  | Per Capita Impressions | 0 |  | 0 |  | 0 |  | 0 |  | 0 |  |  |  |  |  |  |  |  |  |  |  | 0 |  | 0 |  | 0 |  | 0 |  | 0 |  |
