# Supplementary material for: Tobacco and alcohol content in soap operas broadcast on UK television: a content analysis and population exposure
Source: J Public Health (Oxf). 2020 Jul 1;43(3):595–603. doi: 10.1093/pubmed/fdaa091 (PMC8458014; doi:10.1093/pubmed/fdaa091)
Supplement: soaps_sup2_alc_fdaa091 [file soaps_sup2_alc_fdaa091.docx]

| Episode | | November | | | | | | | | | | December | | | | | | | | | | January | | | | | | | | | |
| --- | --- | --- | --- | --- | --- | --- | --- | --- | --- | --- | --- | --- | --- | --- | --- | --- | --- | --- | --- | --- | --- | --- | --- | --- | --- | --- | --- | --- | --- | --- | --- |
|  |  | 5^th^ Part 1 | 5^th^ Part 2 | 6^th^ Part 1 | 6^th^ Part 2 | 7^th^ Part 1 | 7^th^ Part 2 | 8^th^ Part 1 | 8^th^ Part 2 | 9^th^ Part 1 | 9^th^ Part 2 | 3^rd^ Part 1 | 3^rd^ Part 2 | 4^th^ Part 1 | 4^th^ Part 2 | 5^th^ Part 1 | 5^th^ Part 2 | 6^th^ Part 1 | 6^th^ Part 2 | 7^th^ Part 1 | 7^th^ Part 2 | 7^th^ Part 1 | 7^th^ Part 2 | 8^th^ Part 1 | 8^th^ Part 2 | 9^th^ Part 1 | 9^th^ Part 2 | 10^th^ Part 1 | 10^th^ Part 2 | 11^th^ Part 1 | 11^th^ Part 2 |
| Eastenders | Proportion viewership (000s) | 0.096 |  | 0.098 |  |  |  | 0.096 |  | 0.092 |  | 0.093 |  | 0.099 |  |  |  | 0.093 |  | 0.089 |  |  |  | 0.086 |  |  |  | 0.101 |  | 0.097 | 0.082 |
|  | Gross Impressions (Million) | 12.710 |  | 58.406 |  |  |  | 38.196 |  | 67.551 |  | 123.660 |  | 118.132 |  |  |  | 12.343 |  | 53.312 |  |  |  | 0 |  |  |  | 33.833 |  | 25.676 | 5.504 |
|  | Per Capita Impressions | 0.191 |  | 0.880 |  |  |  | 0.575 |  | 1.017 |  | 1.862 |  | 1.779 |  |  |  | 0.186 |  | 0.803 |  |  |  | 0 |  |  |  | 0.510 |  | 0.387 | 0.083 |
|  |  |  |  |  |  |  |  |  |  |  |  |  |  |  |  |  |  |  |  |  |  |  |  |  |  |  |  |  |  |  |  |
| Coronation Street | Proportion Viewership (000s) | 0.089 | 0.085 |  |  | 0.085 | 0.078 |  |  | 0.090 | 0.086 | 0.082 | 0.083 |  |  | 0.080 | 0.074 |  |  | 0.081 | 0.079 | 0.086 | 0.082 |  |  | 0.084 | 0.082 |  |  | 0.086 | 0.083 |
|  | Gross Impressions (Million) | 76.840 | 62.140 |  |  | 67.488 | 62.068 |  |  | 35.966 | 74.296 | 43.564 | 55.313 |  |  | 21.477 | 58.684 |  |  | 16.096 | 84.011 | 28.580 | 27.271 |  |  | 16.728 | 54.518 |  |  | 34.260 | 56.145 |
|  | Per Capita Impressions | 1.157 | 0.936 |  |  | 1.016 | 0.935 |  |  | 0.541 | 1.119 | 0.656 | 0.833 |  |  | 0.323 | 0.884 |  |  | 0.242 | 1.265 | 0.430 | 0.410 |  |  | 0.252 | 0.821 |  |  | 0.516 | 0.846 |
|  |  |  |  |  |  |  |  |  |  |  |  |  |  |  |  |  |  |  |  |  |  |  |  |  |  |  |  |  |  |  |  |
| Emmerdale | Proportion Viewership (000s) | 0.078 |  | 0.074 |  | 0.075 |  | 0.072 | 0.069 | 0.068 |  | 0.073 |  | 0.071 |  | 0.075 |  | 0.070 | 0.070 | 0.070 |  | 0.075 |  | 0.073 |  | 0.074 |  | 0.072 | 0.074 | 0/078 |  |
|  | Gross Impressions (Million) | 25.990 |  | 19.550 |  | 19.996 |  | 62.101 | 50.292 | 46.506 |  | 14.572 |  | 14.191 |  | 39.882 |  | 32.463 | 55.475 | 23.470 |  | 24.790 |  | 33.822 |  | 34.246 |  | 33.614 | 34.370 | 41.298 |  |
|  | Per Capita Impressions | 0.391 |  | 0.294 |  | 0.301 |  | 0.935 | 0.757 | 0.700 |  | 0.219 |  | 0.214 |  | 0.601 |  | 0.489 | 0.835 | 0.353 |  | 0.373 |  | 0.509 |  | 0.516 |  | 0.506 | 0.518 | 0.622 |  |
|  |  |  |  |  |  |  |  |  |  |  |  |  |  |  |  |  |  |  |  |  |  |  |  |  |  |  |  |  |  |  |  |
| Hollyoaks | Proportion Viewership (000s) | 0.012 |  | 0.011 |  | 0.013 |  | 0.014 |  | 0.013 |  |  |  | 0.012 |  | 0.010 |  | 0.012 |  | 0.010 |  | 0.013 |  | 0.014 |  | 0.013 |  | 0.015 |  | 0.012 |  |
|  | Gross Impressions (Million) | 4.916 |  | 3.778 |  | 1.664 |  | 1.866 |  | 6.752 |  |  |  | 1.575 |  | 3.413 |  | 2.481 |  | 2.612 |  | 2.540 |  | 2.880 |  | 0.867 |  | 1.934 |  | 5.539 |  |
|  | Per Capita Impressions | 0.074 |  | 0.057 |  | 0.025 |  | 0.028 |  | 0.102 |  |  |  | 0.024 |  | 0.051 |  | 0.037 |  | 0.039 |  | 0.038 |  | 0.043 |  | 0.013 |  | 0.029 |  | 0.083 |  |
|  |  |  |  |  |  |  |  |  |  |  |  |  |  |  |  |  |  |  |  |  |  |  |  |  |  |  |  |  |  |  |  |
| Neighbours | Proportion Viewership (000s) | 0.011 |  | 0.011 |  | 0.012 |  | 0.011 |  | 0.008 |  | 0.011 |  | 0.010 |  | 0.010 |  | 0.010 |  | 0.011 |  | 0.012 |  | 0.012 |  | 0.010 |  | 0.010 |  | 0.011 |  |
|  | Gross Impressions (Million) | 0 |  | 5.685 |  | 2.301 |  | 7.021 |  | 1.641 |  | 6.070 |  | 3.477 |  | 3.159 |  | 0.682 |  | 2.857 |  | 5.511 |  | 4.707 |  | 3.461 |  | 4.713 |  | 8.122 |  |
|  | Per Capita Impressions | 0 |  | 0.086 |  | 0.035 |  | 0.106 |  | 0.025 |  | 0.091 |  | 0.052 |  | 0.048 |  | 0.010 |  | 0.043 |  | 0.083 |  | 0.071 |  | 0.052 |  | 0.071 |  | 0.122 |  |
|  |  |  |  |  |  |  |  |  |  |  |  |  |  |  |  |  |  |  |  |  |  |  |  |  |  |  |  |  |  |  |  |
| Home and Away | Proportion Viewership (000s) | 0.009 |  | 0.008 |  | 0.008 |  | 0.008 |  | 0.008 |  |  |  |  |  |  |  |  |  |  |  | 0.009 |  | 0.009 |  | 0.008 |  | 0.008 |  | 0.008 |  |
|  | Gross Impressions (Million) | 2.980 |  | 0.554 |  | 1.637 |  | 1.125 |  | 2.082 |  |  |  |  |  |  |  |  |  |  |  | 2.322 |  | 1.236 |  | 2.563 |  | 0 |  | 0 |  |
|  | Per Capita Impressions | 0.045 |  | 0.008 |  | 0.025 |  | 0.017 |  | 0.031 |  |  |  |  |  |  |  |  |  |  |  | 0.035 |  | 0.019 |  | 0.038 |  | 0 |  | 0 |  |
